# *SULT1A1 *genotype, active and passive smoking, and breast cancer risk by age 50 years in a German case–control study

**DOI:** 10.1186/bcr976

**Published:** 2005-01-14

**Authors:** Carmen Lilla, Angela Risch, Silke Kropp, Jenny Chang-Claude

**Affiliations:** 1 Division of Clinical Epidemiology, German Cancer Research Center, Heidelberg, Germany; 2 Division of Toxicology and Cancer Risk Factors, German Cancer Research Center, Heidelberg, Germany

**Keywords:** breast cancer, polymorphism, smoking, sulfotransferase

## Abstract

**Introduction:**

Sulfotransferase 1A1 (encoded by *SULT1A1*) is involved in the metabolism of procarcinogens such as heterocyclic amines and polycyclic aromatic hydrocarbons, both of which are present in tobacco smoke. We recently reported a differential effect of *N*-acetyltransferase (*NAT*) 2 genotype on the association between active and passive smoking and breast cancer. Additional investigation of a common *SULT1A1 *genetic polymorphism associated with reduced enzyme activity and stability might therefore provide deeper insight into the modification of breast cancer susceptibility.

**Methods:**

We conducted a population-based case–control study in Germany. A total of 419 patients who had developed breast cancer by age 50 years and 884 age-matched control individuals, for whom risk factor information and detailed smoking history were available, were included in the analysis. Genotyping was performed using a fluorescence-based melting curve analysis method. Multivariate logistic regression analysis was used to estimate breast cancer risk associated with the *SULT1A1 *Arg^213^His polymorphism alone and in combination with *NAT2 *genotype in relation to smoking.

**Results:**

The overall risk for breast cancer in women who were carriers of at least one SULT1A1*2 allele was not significantly different from that for women with the SULT1A1*1/*1 genotype (adjusted odds ratio 0.83, 95% confidence interval 0.66–1.06). Risk for breast cancer with respect to several smoking variables did not differ substantially between carriers of the *2 allele and noncarriers. However, among NAT2 fast acetylators, the odds ratio associated with passive smoking only (3.23, 95% confidence interval 1.05–9.92) was elevated in homozygous carriers of the SULT1A1*1 allele but not in carriers of the SULT1A1*2 allele (odds ratio 1.28, 95% confidence interval 0.50–3.31).

**Conclusion:**

We found no evidence that the *SULT1A1 *genotype in itself modifies breast cancer risk associated with smoking in women up to age 50 years. In combination with NAT2 fast acetylator status, however, the SULT1A1*1/*1 genotype might increase breast cancer risk in women exposed to tobacco smoke.

## Introduction

Epidemiologic evidence linking cigarette smoking to increased risk for development of breast cancer is mounting (for review [1,2]). In addition, findings from both epidemiology and molecular biology indicate that there is differential susceptibility within the population to development of malignant neoplasms following exposure to certain xenobiotics because of polymorphisms in genes that encode metabolizing enzymes.

Previously, we reported a differential effect of *N*-acetyltransferase (*NAT*) 2 genotype on the association between active and passive smoking and breast cancer risk [3]. The identification of passive smoking as a breast cancer risk factor, particularly for fast acetylators, implied that heterocyclic aromatic amines (HCAs) are among the responsible carcinogens. HCAs are particularly abundant in side-stream tobacco smoke [4] and are activated by *O*-acetylation catalyzed by NATs [5]. Because sulfotransferase (SULT)1A1 (encoded by *SULT1A1*) is also involved in the metabolism of pro-carcinogens from tobacco smoke, the additional investigation of a common polymorphism in the *SULT1A1 *gene might provide deeper insight into the modification of susceptibility to breast cancer.

The SULT1A1 enzyme is generally associated with detoxification of xenobiotic compounds and has been implicated in oestrogen metabolism. However, Glatt and coworkers [6,7] showed that several substances can be activated by the conjugation reaction with SULT1A1, among which are pro-carcinogens such as polycyclic aromatic hydrocarbons and HCAs, both of which are present in tobacco smoke [8,9].

In contrast to earlier assumptions [10], there is increasing evidence that the SULT1A1 enzyme apparently does not play an important role in oestrogen metabolism *in vivo*. Results from *in vitro *studies showed that only the SULT1E1 enzyme is capable of the sulfonation of oestradiol, oestrone and catecholestrogens at physiologically relevant concentrations [11,12]. For instance, Adjei and Weinshilboum [11] showed that K_m _values for the sulfonation of oestradiol with SULT1A1 were in the micromolar range, which is clearly above physiological concentrations, whereas the K_m _value for SULT1E1 was considerably lower (0.029 ± 0.01 μmol/l).

Large interindividual variations in the biochemical and metabolic properties of the SULT1A1 enzyme have been observed that can partly be explained by a G to A polymorphism at nucleotide 638 (Arg^213^His), referred to as the *2 allele. The *2 allele has been associated with lower activity and lower thermal stability of the SULT1A1 enzyme [13,14], and thus reduced bioactivation of mutagens [6].

Thus far, three case–control studies that investigated the association between *SULT1A1 *genotype and breast cancer risk [15-17] have been reported. Results were not consistent and the effect of smoking was not considered in any of those case–control studies. Saintot and coworkers [18] recently reported a positive interaction between smoking and the variant allele for *SULT1A1 *with respect to breast cancer risk in a case-only study.

We conducted the present study to elucidate the potential role of *SULT1A1 *genotype alone and in combination with *NAT2 *genotype as a modifier of susceptibility to breast cancer associated with exposure to tobacco smoke among predominantly premenopausal women.

## Methods

### Study population

The present study is based on a case–control study that is described in greater detail elsewhere [19,20]. In brief, between January 1992 and December 1995 a population-based case–control study on breast cancer was conducted in two regions (Rhein-Neckar-Odenwald and Freiburg regions) in southern Germany. Women with a diagnosis of *in situ *or invasive breast cancer were identified by surveying all of the hospitals that serve the two study regions. Women were eligible for inclusion in the study if they spoke German, if they lived in the study region and if the neoplasm was diagnosed before their 51st birthday. During the period of study 1020 women were identified, of whom 1005 were alive at the time of identification. Of the living, eligible patients, 706 (70.2%) completed a self-administered questionnaire. For every patient, two controls were selected randomly from lists of female residents obtained by the population registries of the study regions and matched according to exact age and residence. Of the 2257 eligible control individuals who were contacted by letter, 1381 (61.2%) participated in the study. After giving written informed consent, all participants completed a self-administered questionnaire and were asked to provide a blood sample. The study is in compliance with the Declaration of Helsinki and was reviewed and approved by the ethics committee of the University of Heidelberg.

The study participants were re-contacted in August 1999 and were invited to participate in a computer-assisted telephone interview to assess comprehensively their history of active and passive smoking [20]. Of the original study population, 66.3% of cases and 78.9% of controls took part in this additional investigation. In short, women were asked when they began smoking, the type of product, the amount and frequency of tobacco usage, the intensity of inhalation, and the date of cessation or changes in their smoking habits. Exposure to passive smoking was assessed in childhood, in the adult household and at work. For passive smoking in adult life, women who had lived with a smoking partner were asked the onset, end, or changes to smoking exposure, daily amount and type of product smoked, and number of hours and days of passive exposure. For childhood exposure as well as exposure at work and that due to other household members, questions pertained to number of smokers living in the household, onset of exposure, and the number of hours and days of smoke exposure that the participant experienced in the presence of each smoking person. All information was truncated at the reference date, which was the date of diagnosis for patients and the date of recruitment for control individuals.

Menopausal status was defined as the reported state at half a year before the reference date. The status of women with previous hysterectomy not accompanied by bilateral oophorectomy was not ascertainable and therefore classified as unknown. Because the study participants were all aged 50 years or younger, these women were included in the analysis restricted to the subgroup with premenopausal status.

Blood samples were available for 95% of cases and 82% of controls in the original study population. This analysis was restricted to women who had either both (97.8%) or at least one parent of German nationality (2.2%) in order to achieve ethnic homogeneity of the study population. In total, 419 patients with breast cancer and 884 control individuals, for whom full genotype information and detailed history of tobacco smoke exposure were available, were included in the analysis.

### Genotyping

DNA was extracted from EDTA blood samples using a standard method based on salt precipitation. *SULT1A1*-specific primers and hybridization probes were used to detect G638A in exon 7. The primers for DNA amplification were previously described by Coughtrie and coworkers [21]. As sensor and anchor probes, we used LCRed640-CAgggAgCgCCCCACAA-p and gAACCATgAAgTCCACggTCTCCTCT-x, respectively. PCR and melting curve analyses were performed in 10 μl volumes in glass capillaries (Roche Diagnostics, Mannheim, Germany) using the following: 1× PCR buffer, 2.5 mmol/l MgCl_2_, 200 μmol/l dNTPs, 0.1% bovine serum albumin, 0.5 U Taq polymerase, 0.15 μmol/l of each probe (TIB MOLBIOL, Berlin), 1 μmol/l of the sense primer (CF) and 0.1 μmol/l of the reverse primer (CR; asymmetric PCR). Approximately 10 ng gDNA was used as a template. The cycling conditions were as follows: initial denaturation at 95°C for 2 min followed by 45 cycles of denaturation at 95°C for 0 s, annealing at 63°C for 5 s and elongation at 72°C for 10 s, with a ramping rate of 20°C/s.

Melting curve analyses were performed with an initial denaturation at 95°C for 10 s, 20 s at 40°C, followed by slow heating of the samples to 80°C with a ramping rate of 0.1°C/s and continuous fluorescence detection. The melting curves were converted to melting peaks by plotting the negative derivatives of fluorescence against temperature (–dF/dT). Melting peaks were mostly unambiguous, but certain samples exhibited abnormal peaks due to two rare silent genetic polymorphisms, one covered by the sensor (G645A [Leu215]) and another covered by the anchor probe (G654A [Glu218]). Forty-eight such samples were additionally digested with *Hha*I [15], allowing unambiguous genotyping at position 638. A further 160 samples selected randomly for quality control exhibited no discrepancies between genotyping results obtained with both methods. Two additional rare genetic variants of the *SULT1A1 *gene (i.e. the *3 [Met^223^Val] and *4 [Arg^37^Gly] alleles), which have been observed in Caucasian populations with allele frequencies of 0.01 and 0.003, respectively, were not accounted for in the present study [22].

Detection of polymorphic sites in the *NAT2 *gene was also carried out by capillary-based PCR followed by melting curve analysis. The method was described in detail previously [3].

### Statistical analysis

The association between active/passive smoking and breast cancer by *SULT1A1 *genotype was assessed by multivariate conditional logistic regression analysis. We computed maximum likelihood estimates for the odds ratios (ORs) and their 95% confidence intervals (CIs) using the PHREG procedure of the statistical software package SAS release 8.2 (SAS Institute, Cary, NC, USA).

'Ever active smoking' was defined as having smoked more than 100 cigarettes in one's life. Among ever active smokers, women were termed current smokers if they had smoked regularly within the year preceding the interview; otherwise, they were classified as former smokers. If women were on average exposed to passive smoke for more than 1 hour/day for at least 1 year, then they were defined as ever passive smokers. The average exposure was obtained by multiplying the average hours/day by the duration in years for each exposure phase and dividing the sum over all phases separately for childhood and adulthood by the total years of passive exposure. Missing data on hours/day for 7.7% of cases and 5.7% of controls were replaced with the mean hours/day of exposed controls for the particular source of exposure. A detailed description of the quantification of lifetime exposure to passive smoke can be found elsewhere [20].

In the multivariate model, we included several relevant variables that influence breast cancer risk, such as first-degree family history of breast cancer, total duration of breastfeeding, body mass index, average daily alcohol intake, education level, number of full-term pregnancies and menopausal status. Variables that did not change the estimates substantially, such as study region or age at menarche, were not adjusted for in the analyses presented here. Statistical interaction between genotype and smoking variables was tested by using multiplicative interaction terms and evaluated using the likelihood ratio test. We performed the multivariate analyses with stratification in 5-year age groups to ensure sufficient numbers of subjects in the subgroups for genotypes and smoking characteristics.

## Results

The women included in the present study, for whom a comprehensive history of active and passive smoking was available, closely resemble the original study population with respect to the distributions of several sociodemographic characteristics and putative risk factors, such as age, family history of breast cancer, body mass index, education level, parity, menopausal status, alcohol consumption, smoking and breastfeeding (data not shown).

Selected characteristics of the present study population are summarized in Table 1. The mean (± standard deviation) age for breast cancer patients was 42.9 ± 5.5 years and that for control individuals was 42.7 ± 5.6 years. The frequency of the SULT1A1*2 allele was 0.33 among cases and 0.35 among controls (0.32 and 0.34 in the original population). Of cases and control individuals, 52.7% and 57.7%, respectively, were carriers of at least one SULT1A1*2 allele. The distribution of *SULT1A1 *genotypes was in Hardy–Weinberg equilibrium (*P *= 0.92 for control individuals, *P *= 0.09 for cases).

The overall risk for breast cancer among carriers of the SULT1A1*2 allele was not significantly different from that in women with the SULT1A1*1/*1 genotype (adjusted OR 0.83, 95% CI 0.66–1.06). The distributions of potential risk factors, such as first-degree family history of breast cancer, body mass index, alcohol consumption, menopausal status, parity and breastfeeding, were similar in carriers and noncarriers of the SULT1A1*2 allele. There was also no major effect of *SULT1A1 *genotype in combination with NAT2 acetylator status on breast cancer risk (data not shown).

We assessed the effect of *SULT1A1 *genotype on the association between smoking and breast cancer risk, initially comparing ever active smokers with nonsmokers (i.e. passive-only smokers were included in the reference group). The ORs for variables such as smoking status (current or former active smoker), duration and pack-years of smoking did not differ by *SULT1A1 *genotype (data not shown). We then considered a separate category of only passively exposed women, with a reference group comprising women with neither active nor passive cigarette smoke exposure (Table 2). Associations of breast cancer risk with smoking variables were apparent, but the risk estimates were similar for carriers and for noncarriers of the SULT1A1*2 allele. In the analysis of passive smoking among never active smokers, we observed a tendency toward higher ORs in women with the SULT1A1*1/*1 genotype compared with carriers of the SULT1A1*2 allele (Table 2). The test for interaction between *SULT1A1 *genotype and passive smoking was not statistically significant (*P *= 0.6).

We investigated the combined effect of *SULT1A1 *and *NAT2 *genotype with respect to smoking, and observed elevated ORs associated with passive smoking only (OR 3.23, 95% CI 1.05–9.92) in NAT2 fast acetylators with the SULT1A1*1/*1 genotype but not in NAT2 fast acetylators carrying the SULT1A1*2 allele (Table 3). There was also a difference in OR for 11 or more pack-years of active smoking by *SULT1A1 *genotype, but the risk estimates were not significant. The test for interaction between *SULT1A1 *genotype and active/passive smoking among NAT2 fast acetylators did not reach statistical significance (*P *= 0.4). Among NAT2 slow acetylators, risk estimates for active and passive smoking did not differ by *SULT1A1 *genotype.

The results were generally similar when the analysis was restricted to the subgroup of women with premenopausal status, although the confidence intervals were wider. With regard to the differential effect of *SULT1A1 *and *NAT2 *genotype, the OR for passive smoking was 2.24 (95% CI 0.68–7.35) in NAT2 fast acetylators with the SULT1A1*1/*1 genotype and 1.03 (95% CI 0.39–2.71) in fast acetylators carrying the SULT1A1*2 allele.

## Discussion

Our data do not suggest a strong influence of *SULT1A1 *genotype alone or in combination with *NAT2 *on the risk for breast cancer. There is no clear evidence that the *SULT1A1 *Arg^213^His single nucleotide polymorphism investigated in this study in itself is an important effect modifier of breast cancer risk associated with active/passive smoking among women up to age 50 years.

Differences in risk estimates for carriers and noncarriers of the SULT1A1*2 allele associated with smoking were apparent among NAT2 fast acetylators but not among slow acetylators. The observed estimates indicated that fast acetylators with the SULT1A1*1/*1 genotype were at higher risk for breast cancer than were carriers of the SULT1A1*2 allele when exposed to tobacco smoke, with a particularly prominent increase in risk for passive smokers versus never active/passive smokers.

We cannot rule out the possibility that the observed risk elevation for SULT1A1*1/*1 among fast acetylators was due to chance, because confidence intervals were wide for the combined analysis of genotypes. However, it seems biologically plausible that the combination of *NAT2 *and *SULT1A1 *'fast' genotypes is unfavourable. Both enzymes have been shown to be capable of bioactivating several pro-carcinogens. NAT2 is thought to play a major role in the activation of *N*-hydroxy derivatives of HCAs by *O*-acetylation [5]. The sulfonation of a variety of xenobiotics or their metabolites, such as polycyclic aromatic hydrocarbons, HCAs and aromatic amines, can lead to short-lived conjugates that may react with DNA and other cellular nucleophiles [23]. Studies that investigated the genotype–phenotype correlation for *SULT1A1 *clearly indicate that the SULT1A1*2 allele is associated with decreased catalytic activity of the respective allozyme as compared with the sulfonation activity of the wild-type SULT1A1 enzyme [6,13,14]. Consequently, in individuals with this genotype combination, reactive metabolites from acetylation and sulfonation might accumulate and lead to greater DNA damage and increase tumourigenesis.

For instance, DNA adducts of 2-amino-3-methylimidazo [4,5-*f*]quinoline (IQ) and 2-amino-1-methyl-6-phenylimidazo [4,5-*b*]pyridine (PhIP) have been detected in human breast milk [24]. These HCAs, which are also present in tobacco smoke, have been classified as probably and possibly carcinogenic to humans, respectively [25]. In mutagenicity assays after heterologous expression of NAT2 and SULT1A1 in *Salmonella typhimurium*, *N*-hydroxy-IQ was found to be efficiently activated by NAT2 whereas *N*-hydroxy-PhIP was specifically activated by the SULT1A1 enzyme [26]. Likewise, mutagenicity of 2-amino-3-methyl-9*H*-pyrido [2,3-*b*]indole, another abundant HCA, was strongly enhanced in a *Salmonella typhimurium *strain expressing SULT1A1 [27].

Consistent with a role of greater bioactivation of HCAs associated with SULT1A1*1 rather than SULT1A1*2 in carcinogenesis are previous reports of greater risk for breast cancer and prostate cancer associated with intake of well done red meat [14,15]. In accord with this notion are the findings of three studies that investigated the formation of DNA adducts of heterocyclic and aromatic amines [28-30]. All of them found a tendency toward a higher capacity for adduct formation for the SULT1A1*1 enzyme as compared with the *2 allozyme, which is in agreement with a more efficient activation of several pro-mutagens by SULT1A1*1 than by the *2 allelic variant reported by Glatt and coworkers [6].

Two previous studies [15,17] reported an increased risk for breast cancer associated with the SULT1A1*2 allele *per se*. Risk estimates were statistically significant only in the study conducted by Zheng and coworkers [15], a nested case–control study in postmenopausal women, which found an 80% elevated risk for homozygous carriers of the variant allele. We found no association with the SULT1A1*2 variant although our study had 93% power to detect an OR of equivalent magnitude at a significance level of α = 0.05.

In a recent case-only study, an interaction between the *SULT1A1 *polymorphism and tobacco smoke exposure with an OR for interaction of 2.55 (95% CI 1.21–5.36) for current smokers carrying the *2 allele was found [18]. Results from our case–control study did not provide an indication for such a strong interaction between *SULT1A1 *genotype and smoking. Accordingly, we failed to detect a significant interaction in a case-only analysis of our data, although the power of our study is similar and the precondition of independence between genotype and exposure in the general population was fulfilled. The ORs (95% CIs) for interaction were 1.04 (0.50–2.14) for passive smoking only, 1.34 (0.63–2.86) for former smoking, and 1.15 (0.55–2.39) for current smoking.

We feel confident that our study population is representative of the general German population. The observed allele frequencies for *SULT1A1 *are in accordance with previous studies conducted in Caucasian populations [31] and the *SULT1A1 *genotype distribution did not deviate from Hardy–Weinberg equilibrium. Re-contacting the study participants for the telephone interview might have introduced selection bias. However, the participants in the present study closely resemble the original study population with regard to the distributions of relevant characteristics. Also, we do not believe that recall bias is a major concern because smoking was not known to be associated with breast cancer at the time of the interviews, and the correlation of reported active smoking between the original study and the present study is high [20]. Moreover, previous studies showed that the validity for self-reported active smoking, as well as passive smoke exposure, is high and nondifferential in cases and controls [32,33].

Although *in vitro *data suggest that SULT1A1 may not play an important role at physiologically relevant oestrogen concentrations [11,12], the question regarding whether *SULT1A1 *genotype actually has an effect on oestrogen metabolism *in vivo *deserves further study. Concerning the expression of SULT1A1 and SULT1E1 enzymes, for instance, there is some controversy in the literature. Falany and coworkers [34,35] observed SULT1E1 expression in normal breast epithelial cells, whereas Williams and coworkers [36] reported that only SULT1A1 was expressed at detectable levels. Because we cannot definitely rule out a potential role of SULT1A1 in the metabolism of oestrogens, we analyzed our data also with respect to use of oral contraceptives and various reproductive factors that may alter exposure to oestrogens. The results did not provide any indication for a modification of breast cancer risk related to oestrogens by *SULT1A1 *genotype (data not shown) and corroborate recent evidence indicating that sulfonation of oestrogens catalyzed by SULT1A1 is less relevant in normal breast tissue in physiological conditions [11,12].

The inconsistent findings of previous studies, which also considered the possible involvement of the *SULT1A1 *gene in other cancer sites (summarized by Glatt and Meinl [23]), and the broad substrate specificity of the SULT1A1 enzyme indicate the complexity of the issue. Elucidation of the potential effects of *SULT1A1 *genotype on a hormone-related cancer, such as breast cancer, is rendered more complicated by the fact that smoking may alter oestrogen levels in the body [37-40]. Moreover, and as suggested by our findings, it is possible that *SULT1A1 *genotype only exerts a detectable effect in combination with other genes, not to mention several polymorphic genes that are involved in oestrogen metabolism. Further determinants such as varying levels of enzyme expression or enzyme induction, which cannot easily be assessed in epidemiological studies, might also be of importance. Nevertheless, we cannot exclude that, because of limitations in statistical power, we were unable to detect a potential weak or moderate association or interaction between *SULT1A1 *genotype, smoking and breast cancer, independent of *NAT2 *genotype.

## Conclusion

In summary, the results of our study do not suggest that there is a strong association between the *SULT1A1 *Arg^213^His genetic polymorphism and risk for breast cancer in women who had developed breast cancer by age 50 years. We did not find any evidence for a significant interaction of *SULT1A1 *with smoking. The SULT1A1*1/*1 genotype in combination with NAT2 fast acetylator status, however, appeared to increase breast cancer risk in women exposed to tobacco smoke. Hence, further biochemical investigations and large molecular epidemiologic studies are required to evaluate the effects of multiple genes and exposures on susceptibility to breast cancer.

## Abbreviations

CI = confidence interval; HCA = heterocyclic aromatic amine; IQ = 2-amino-3-methylimidazo [4,5–*f*]quinoline; NAT = *N*-acetyltransferase; OR = odds ratio; PCR = polymerase chain reaction; PhIP = 2-amino-1-methyl-6-phenylimidazo [4,5–*b*]pyridine; SULT = sulfotransferase.

## Competing interests

The author(s) declare that they have no competing interests.

## Authors' contributions

CL performed the statistical analysis and drafted the manuscript. AR was responsible for the genotyping assays, and contributed to study design and manuscript preparation. SK conducted the re-contacting of study participants in 1999 and participated in the statistical analyses. JCC conceived the study and supervised the project. All authors read and approved the final manuscript.

## Figures and Tables

**Table 1:** Selected characteristics of breast cancer patients and controls

Characteristic	Cases (*n *= 419)	Controls (*n *= 884)
Mean age (years)	42.9	42.7
Mean body mass index	23.9	24.3
First-degree family history of breast cancer	62 (14.8%)	53 (6.0%)
Menopausal status		
Premenopausal	325 (77.6%)	718 (81.2%)
Postmenopausal	25 (6.0%)	49 (5.5%)
Unknown	69 (16.5%)	117 (13.2%)
*SULT1A1 *genotype		
*1/*1	198 (47.3%)	374 (42.3%)
*1/*2	169 (40.3%)	403 (45.6%)
*2/*2	52 (12.4%)	107 (12.1%)
*NAT2 *genotype^a^		
Fast acetylator	177 (42.2%)	351 (39.7%)
Slow acetylator	242 (57.8%)	533 (60.3%)

^a^*NAT2 *fast acetylators are carriers of at least one *4 (wild-type) allele, based on detection of known genetic polymorphisms at nucleotide positions 481, 590, 803 and 857; slow acetylators are carriers of two variant alleles [3].

**Table 2:** Odds ratios for breast cancer associated with smoking variables stratified by *SULT1A1 *genotype

	SULT1A1*1/*1 genotype			SULT1A1*2 allele carrier^a^		
Variable	Cases (*n *= 198)	Controls (*n *= 374)	OR^b ^(95% CI)	Cases (*n *= 221)	Controls (*n *= 510)	OR^b ^(95% CI)

Never active	86	159	1 (ref)	88	209	1 (ref)
Ever active	112	215	0.92 (0.63–1.34)	133	301	1.01 (0.73–1.42)
Never active/passive^c, d^	20	56	1 (ref)	19	57	1 (ref)
Former active	44	94	1.23 (0.64–2.36)	59	157	1.11 (0.60–2.06)
Current active	68	121	1.44 (0.76–2.71)	74	144	1.51 (0.82–2.78)
Average cigarettes per day^c, e^						
>0–9	42	106	1.06 (0.55–2.03)	52	154	0.99 (0.54–1.84)
10–19	56	73	2.12 (1.10–4.07)	60	99	1.75 (0.94–3.25)
20+	14	34	1.05 (0.45–2.46)	20	48	1.09 (0.51–2.32)
Duration of smoking^c, d^						
1–15 years	36	80	1.19 (0.61–2.34)	46	145	0.97 (0.52–1.82)
16+ years	76	135	1.44 (0.77–2.69)	87	156	1.62 (0.89–2.95)
Age at first cigarette^c, d^						
9–15 years	21	47	1.09 (0.51–2.35)	20	63	0.84 (0.40–1.79)
16–18 years	56	109	1.33 (0.70–2.52)	70	145	1.42 (0.77–2.62)
19+ years	35	59	1.51 (0.75–3.05)	43	93	1.42 (0.74–2.72)
Pack-years^c, e^						
>0–10	59	129	1.22 (0.65–2.27)	76	191	1.15 (0.64–2.07)
11+	53	84	1.77 (0.91–3.42)	56	110	1.43 (0.77–2.67)
Passive smoking^f^						
No	20	57	1 (ref)	19	57	1 (ref)
Yes	66	102	1.69 (0.89–3.21)	69	152	1.40 (0.74–2.64)
Duration of passive exposure in adulthood^f, g, h^						
1–13 years	29	40	1.91 (0.91–4.00)	33	70	1.47 (0.72–2.98)
14+ years	32	47	1.66 (0.79–3.48)	29	61	1.47 (0.70–3.10)
Cumulative lifetime exposure (in hours/day-years)^f, g, i^						
1–55	22	49	1.13 (0.52–2.38)	37	76	1.76 (0.89–3.50)
56+	43	52	2.12 (1.06–4.25)	32	71	1.39 (0.68–2.83)

^a^Includes SULT1A1*1/*2 and SULT1A1*2/*2 genotypes. ^b^Odds ratios (ORs) stratified for age by 5-year intervals; additional adjustment was made for first-degree family history (yes/no), breastfeeding (total number of months) and body mass index (weight [kg]/ height [m]^2^) as continuous variables, parity (0, 1–2, 3+ children), alcohol consumption (0, 1–18, 19+ g/day), menopausal status (premenopausal, postmenopausal, unknown) and education (low, intermediate, high) as categorical variables. ^c^Reference group comprises never active/never passive smokers; category of passive smokers included in the models. ^d^Data missing for one control individual. ^e^Data missing for three control individuals and one case. ^f^Ever active smokers are excluded from the analysis; reference group consists of women not exposed to passive smoke. ^g^Dichotomization according to median of nonsmoking control individuals. ^h^Category of subjects only exposed during childhood is included in the model. ^i^Sum of hours/day-years for the sources partner, work and childhood, whereby childhood hours/day-years were divided by the number of smokers to avoid overlapping of exposures; data missing for one case and six control individuals.

**Table 3:** Odds ratios for breast cancer associated with smoking stratified by NAT2 acetylator status and *SULT1A1 *genotype

	SULT1A1*1/*1 genotype			SULT1A1*2 allele carrier^a^			Total		
	
	Cases	Controls	OR^b ^(95% CI)	Cases	Controls	OR^b ^(95% CI)	Cases	Controls	OR^b ^(95% CI)
NAT2 fast acetylator^c^									
Never active/passive	6	20	1 (ref)	8	18	1 (ref)	14	38	1 (ref)
Passive only	30	37	3.23 (1.05–9.92)	37	59	1.28 (0.50–3.31)	67	96	1.95 (0.97–3.91)
1–10 pack-years	22	53	1.26 (0.40–3.95)	30	74	0.76 (0.29–1.96)	52	127	0.98 (0.48–1.98)
11+ pack-years	23	38	2.21 (0.68–7.12)	20	50	0.75 (0.27–2.06)	43	88	1.22 (0.59–2.54)
NAT2 slow acetylator									
Never active/passive	14	33	1 (ref)	11	39	1 (ref)	25	75	1 (ref)
Passive only	36	65	1.35 (0.62–2.91)	32	93	1.18 (0.53–2.66)	68	158	1.25 (0.72–2.16)
1–10 pack-years	37	76	1.24 (0.57–2.69)	46	117	1.34 (0.62–2.92)	83	193	1.24 (0.72–2.12)
11+ pack-years	30	46	1.83 (0.79–4.26)	36	60	2.07 (0.91–4.72)	66	106	1.85 (1.04–3.30)

^a^Includes SULT1A1*1/*2 and SULT1A1*2/*2 genotypes. ^b^Odds ratios (ORs) stratified for age by 5-year intervals; additional adjustment was made for first-degree family history (yes/no), breastfeeding (total number of months) and body mass index (weight [kg]/ height [m]^2^) as continuous variables, parity (0, 1–2, 3+ children), alcohol consumption (0, 1–18, ≥19 g/day), menopausal status (premenopausal, postmenopausal, unknown) and education (low, intermediate, high) as categorical variables. Data missing for one case and three controls. ^c^NAT2 fast acetylators are carriers of at least one *4 (wild-type) allele.
